# Fear of anesthesia for cesarean section among pregnant women: a multicenter cross-sectional study

**DOI:** 10.1186/s13741-023-00353-9

**Published:** 2023-11-24

**Authors:** Ramzi Shawahna, Mohammad Jaber, Iyad Maqboul, Hatim Hijaz, Eman Arjan, Maisa Karaki, Keen Nasser

**Affiliations:** 1 Department of Physiology, Pharmacology and Toxicology, Faculty of Medicine and Health Sciences, An-Najah National University, Nablus, Palestine; 2 Clinical Research Center, An-Najah National University Hospital, Nablus, 44839 Palestine; 3 Department of Medicine, Faculty of Medicine and Health Sciences, An-Najah National University, Nablus, Palestine; 4 An-Najah National University Hospital, An-Najah National University, Nablus, Palestine

**Keywords:** Anesthesia, Fear, Cesarean delivery, Women, Delivery

## Abstract

**Background:**

Fear of anesthesia for cesarean section delivery is an important health issue that should be addressed by anesthesiologists, obstetricians, and other providers of antenatal and perioperative healthcare. This multicenter study was conducted to assess the fear of anesthesia for cesarean section among Palestinian pregnant women and to identify the predictors of high fear.

**Methods:**

The study was conducted in a cross-sectional design among Palestinian pregnant women in different antenatal healthcare clinics. The study participants were recruited using a convenience sampling procedure. The pregnant women were asked to respond to items in a questionnaire.

**Results:**

In this study, a total of 394 pregnant women completed the questionnaires. Of the pregnant women, 280 (71.1%) have had cesarean delivery under anesthesia, and 104 (26.4%) elected cesarean delivery. Multiple linear regression showed that higher fear scores were predicted by having a university degree (*p*-value < 0.001), living in rural areas (*p*-value = 0.007), dissatisfaction with social life (*p*-value = 0.004), satisfaction with religious commitment (*p*-value = 0.001), having had cesarean delivery under anesthesia (*p*-value = 0.005), and preference of cesarean delivery (*p*-value < 0.001).

**Conclusion:**

Fear of anesthesia was prevalent among pregnant women in Palestine. Higher fear could be predicted by education level, place of residence, satisfaction with social life, satisfaction with religious commitment, having had cesarean delivery under anesthesia, and preference for normal delivery. Anesthesiologists, gynecologists and obstetricians, nurses, midwives, and other providers of antenatal and perioperative healthcare services should consider these factors while counseling pregnant women and addressing their fear of anesthesia.

## Background

In today’s obstetrics practice, cesarean sections are one of the most widely performed surgeries in different healthcare systems around the globe (Betran et al. 2021; Martin et al. 2021). Recently, there has been a significant increase in the rates of cesarean section deliveries around the world. According to a recent study conducted by the World Health Organization, cesarean sections account for more than 20% of all childbirths globally (Betran et al. 2021). The same study projected that in 2030, cesarean sections would account for about one-third of all childbirths worldwide. According to the US National Center for Health Statistics, there have been 1,186,397 cesarean section deliveries in the USA in 2019 which accounted for about 32% of all childbirths in the USA (Martin et al. 2021). In some regions of the world, more than 50% of deliveries were cesarean section deliveries (Zhang et al. 2008). Cesarean sections can be performed under general or regional anesthesia (Sumikura et al. 2016; Al-Husban et al. 2021). There are two types of regional anesthesia: epidural and spinal anesthesia.

Fear of anesthesia is an important health issue that has to be addressed by anesthesiologists and other providers of perioperative care (Mavridou et al. 2013). Previous studies have reported that the majority of patients express anxiety and fear once a decision on a surgical intervention has been made until the moment the patients are admitted to the operating room (Valenzuela Millan et al. 2010). In Turkey, the majority of the patients who were interviewed expressed fear of postoperative pain, waking up during surgery, or being sleepy after surgery (Kilinc and Ozer 2017; Ruhaiyem et al. 2016). Similarly, the majority of patients interviewed in Greece also expressed fears and anxiety preoperatively (Mavridou et al. 2013). In the previous studies, the majority of the fears of the patients were attributed to the fear of anesthesia (Mavridou et al. 2013; Celik and Edipoglu 2018). If not appropriately addressed, the fear of patients can complicate anesthesia and other perioperative care provided to the patients (Celik and Edipoglu 2018).

It has been argued that fear of anesthesia can be simply addressed by adequate counseling and providing the patients with complete information about the anesthesia including answering the questions of the patients in an appropriately planned preoperative patient assessment visit (Mavridou et al. 2013; Lozada et al. 2016). During this visit, the anesthesiologists and providers of perioperative care should make every effort to ensure calming the patients and addressing their concerns (Kindler et al. 2005).

In Palestine, little is known about the fear of anesthesia for cesarean section delivery among pregnant women. Therefore, this study was conducted to assess the fear of anesthesia for cesarean section among Palestinian pregnant women. Another aim was to identify the factors that could predict the fear of anesthesia among pregnant women. The findings of this study could be informative to anesthesiologists, gynecologists and obstetricians, nurses, midwives, and other providers of antenatal and perioperative healthcare services.

## Methods

### Study design

This multicenter study was conducted in a cross-sectional design among Palestinian pregnant women. The study was conducted and reported in adherence to Strengthening the Reporting of Observational Studies in Epidemiology (STROBE) guidelines (Vandenbroucke et al. 2007).

### The study population, sample size, and inclusion criteria

The population in this study was pregnant women who were scheduled for cesarean section deliveries and pregnant women who could be potentially scheduled for cesarean section deliveries. The sample size was calculated using Daniel’s formula for a maximal sample size. The sample size was calculated at a 95% confidence interval (95% CI) for a population of more than 20,000 pregnant women and tolerating a margin of error of 5%. The sample size needed for this study was 385 pregnant women.

Women were included if they were pregnant, visiting antenatal healthcare clinics in the West Bank of Palestine, willing to provide informed consent, and willing to answer items in a questionnaire.

### Recruitment and data collection

The study participants were recruited using a convenience sampling procedure from different antenatal healthcare clinics in the West Bank of Palestine. The pregnant women who participated in this study were invited by the researchers who explained the objectives and obtained written informed consent from the women who expressed willingness to participate.

The study tool used in this study was a questionnaire. The questionnaire was developed based on previous studies that were conducted to assess the fear of anesthesia for cesarean section among pregnant women elsewhere (Mavridou et al. 2013; Kilinc and Ozer 2017; Ruhaiyem et al. 2016; Dursun et al. 2011).

### Validity and reliability of the questionnaire

The questionnaire was assessed for face and content validity by a panel of healthcare professionals. The panel included anesthesiologists (*n* = 2), gynecologists and obstetricians (*n* = 3), nurses (*n* = 3), and midwives (*n* = 2). The panelists rated each item in the questionnaire for suitability using a Likert scale of 1–5 (1 = strong disagreement that the item was suitable, 5 = strong agreement that the item was suitable). Items that were rated as suitable by the panelists were included. Conflicting ratings were resolved through discussions and consensus (Shawahna 2021; Shawahna and Awawdeh 2021; Shawahna et al. 2023a).

To assess the questionnaire for readability and comprehension, a pilot test was conducted among 25 women. The women were asked to respond to the questionnaire. The test-retest method was used to ensure the stability of responses over a short period (Shawahna et al. 2023a; Shawahna et al. 2023b). The women were asked to respond to the questionnaire twice. Responses of the women in both rounds were correlated using Pearson’s correlations. It was decided *a priori* that a Pearson’s correlation coefficient of > 0.80 would indicate acceptable stability of scores (Shawahna et al. 2023b). In this study, Pearson’s r was 0.94 which indicated excellent test-retest reliability. The item relatedness (internal consistency) was assessed by Cronbach’s α. It was decided *a priori* that Cronbach’s α > 0.70 would indicate acceptable item relatedness (Shawahna et al. 2023a; Shawahna et al. 2023b). In this study, Cronbach’s α was > 0.70 which indicated good internal consistency of the questionnaire.

### Statistical analysis

Data were entered into IBM SPSS v.21.0 for Windows. Data were expressed as numbers, percentages, and means with their standard deviations (SD). Scores of groups were compared using *t*-tests. Correlations were established using Pearson’s correlation coefficients. A multiple linear regression model was used to investigate the strength of association between the different variables of scores. The variables that were significantly associated with the *t*-tests were retained in the model.

### Ethical considerations

The study was conducted in adherence to the international ethical standards and guidelines followed in scientific research that involves humans. This study was approved by the Institutional Review Board (IRB) of An-Najah National University (An-Najah National University IRB—Jan 21, 2021). The pregnant women provided written informed consent before they responded to the questionnaire.

## Results

### Variables of the women

In this study, 500 pregnant women were invited to participate. Of those, 394 pregnant women completed the questionnaires, giving a response rate of 78.8%. Of the pregnant women, 207 (52.5%) were 30 years or older, 242 (61.4%) had a university education, and 200 (50.8%) lived in urban areas. Of the women, 180 (45.7%) were employed, and 309 (78.4%) expressed satisfaction with their household income. When asked about their social life and religious commitment, 334 (84.8%) and 365 (92.6%) were satisfied, respectively. Of the women, 331 (84.0%) reported a stable marital life, and 318 (80.7%) reported living a happy life. Of the pregnant women, 280 (71.1%) have had cesarean delivery under anesthesia, and 104 (26.4%) elected cesarean delivery. Details of the variables of the women are shown in Table 1.

**Table 1:** Detailed variables of the women

Variable	*n*	%
**Age (years)**		
< 30	187	47.5
≥ 30	207	52.5
**Marital status**		
Currently married	368	93.4
Divorced/Widowed	26	6.6
**Educational level**		
School	152	38.6
University	242	61.4
**Place of residence**		
Rural	194	49.2
Urban	200	50.8
**Employment status**		
Unemployed	214	54.3
Employed	180	45.7
**Self-rated level of satisfaction with household income**		
Not satisfied	85	21.6
Satisfied	309	78.4
**Self-rated level of satisfaction with social life**		
Not satisfied	60	15.2
Satisfied	334	84.8
**Self-rated level of satisfaction with religious commitment**		
Not satisfied	29	7.4
Satisfied	365	92.6
**Self-rated level of marital stability**		
Not stable	63	16.0
Stable	331	84.0
**Self-rated happiness in life**		
Not happy	76	19.3
Happy	318	80.7
**Have had cesarean delivery under anesthesia**		
No	114	28.9
Yes	280	71.1
**Self-declared preference for mode of delivery**		
Normal delivery	290	73.6
Cesarean delivery	104	26.4

### Fear of anesthesia for cesarean delivery among pregnant women

In this study, the pregnant women expressed fear of anesthesia. Of the pregnant women, 307 (77.9%) were afraid of postoperative pain, 248 (62.9%) were afraid of not waking up after surgery, and 238 (60.4%) were afraid of being nauseous postoperatively. Of the women, 240 (60.9%) were afraid of needles and drains, and 217 (55.1%) were afraid of the anesthesiologist not attending to them throughout surgery. Fear of vomiting, being sleepy for hours, and receiving improper postoperative care were expressed by 225 (57.1%), 207 (52.5%), and 248 (62.9%) women, respectively. Fear of unsuccessful anesthesia, waking up during surgery, and being paralyzed because of anesthesia were expressed by 249 (63.2%), 237 (60.1%), and 211 (53.5%) women, respectively. Of the women, 201 (51.0%) were afraid of clarity of thought, and 248 (63.0%) were afraid of admission to the intensive-care unit. In addition, 233 (59.1%) women were afraid of the anesthesiologist not being experienced or qualified, and 241 (61.2%) women were afraid of revealing personal issues under anesthesia. Fear of pregnant women is shown in Table 2.

**Table 2:** Fear of anesthesia for cesarean delivery among pregnant women

		Strongly disagree		Disagree		Neutral		Agree		Strongly agree	
No.	Reason	*n*	%	*n*	%	*n*	%	*n*	%	*n*	%
1	I’m afraid of postoperative pain	1	0.3	45	11.4	41	10.4	168	42.6	139	35.3
2	I’m afraid of not waking up after surgery (fear of death)	8	2.0	72	18.3	66	16.8	106	26.9	142	36.0
3	I’m afraid of being nauseous postoperatively	8	2.0	79	20.1	69	17.5	129	32.7	109	27.7
4	I’m afraid of needles and drains	7	1.8	75	19.0	72	18.3	132	33.5	108	27.4
5	I’m afraid of the anesthesiologist not attending to me throughout the surgery	13	3.3	94	23.9	70	17.8	115	29.2	102	25.9
6	I’m afraid of vomiting postoperatively	8	2.0	91	23.1	70	17.8	129	32.7	96	24.4
7	I’m afraid of being sleepy for hours postoperatively	10	2.5	100	25.4	77	19.5	115	29.2	92	23.4
8	I’m afraid of improper postoperative care	6	1.5	72	18.3	68	17.3	144	36.5	104	26.4
9	I’m afraid of unsuccessful anesthesia (awareness during anesthesia)	8	2.0	72	18.3	65	16.5	145	36.8	104	26.4
10	I’m afraid of waking up during surgery	7	1.8	89	22.6	61	15.5	129	32.7	108	27.4
11	I’m afraid of being paralyzed because of anesthesia	8	2.0	83	21.1	92	23.4	112	28.4	99	25.1
12	I’m afraid of the clarity of thoughts affected by anesthesia	13	3.3	100	25.4	80	20.3	110	27.9	91	23.1
13	I’m afraid of admission to the intensive-care unit	7	1.8	64	16.2	75	19.0	137	34.8	111	28.2
14	I’m afraid of the anesthesiologist not being experienced and qualified enough for my case	8	2.0	73	18.5	80	20.3	121	30.7	112	28.4
15	I’m afraid of revealing personal issues under the influence of anesthesia	13	3.3	57	14.5	83	21.1	91	23.1	150	38.1

When the mean scores were compared, significantly higher fear scores were expressed by the pregnant women who had university degrees (*p*-value = 0.001) and lived in rural areas (*p*-value = 0.008). Similarly, the fear scores were significantly higher for the women who were not satisfied with their social life (*p*-value = 0.013) and those who were satisfied with their religious commitment (*p*-value = 0.001). In addition, significantly higher fear scores were expressed by the pregnant women who have had cesarean delivery under anesthesia (*p*-value = 0.013) and those who preferred normal delivery (*p*-value < 0.001). Details of the mean scores are shown in Table 3.

**Table 3:** The mean fear scores

Variable	*n*	%	Mean	SD	*p*-value
**Age (years)**					
< 30	187	47.5	54.4	13.6	0.768
≥ 30	207	52.5	54.8	13.1	
**Marital status**					
Currently married	368	93.4	54.7	13.5	0.942
Divorced/widowed	26	6.6	54.5	11.5	
**Educational level**					
School	152	38.6	51.9	13.1	0.001
University	242	61.4	56.4	13.2	
**Place of residence**					
Rural	194	49.2	56.5	13.2	0.008
Urban	200	50.8	52.9	13.3	
**Employment status**					
Unemployed	214	54.3	54.9	13.5	0.641
Employed	180	45.7	54.3	13.2	
**Self-rated level of satisfaction with household income**					
Not satisfied	85	21.6	55.5	14.0	0.515
Satisfied	309	78.4	54.4	13.2	
**Self-rated level of satisfaction with social life**					
Not satisfied	60	15.2	58.6	13.9	0.013
Satisfied	334	84.8	53.9	13.1	
**Self-rated level of satisfaction with religious commitment**					
Not satisfied	29	7.4	46.3	11.1	0.001
Satisfied	365	92.6	55.2	13.3	
**Self-rated level of marital stability**					
Not stable	63	16.0	53.1	13.9	0.309
Stable	331	84.0	54.9	13.2	
**Self-rated happiness in life**					
Not happy	76	19.3	53.5	13.3	0.399
Happy	318	80.7	54.9	13.3	
**Have had cesarean delivery under anesthesia**					
No	114	28.9	52.0	11.7	0.013
Yes	280	71.1	55.7	13.8	
**Self-declared preference for mode of delivery**					
Normal delivery	290	73.6	56.2	12.8	< 0.001
Cesarean delivery	104	26.4	50.5	13.9	

The multiple linear regression model showed that fear scores were predicted by having a university degree (*p*-value < 0.001), living in rural areas (*p*-value = 0.007), dissatisfaction with social life (*p*-value = 0.004), satisfaction with religious commitment (*p*-value = 0.001), having had cesarean delivery under anesthesia (*p*-value = 0.005), and preference of cesarean delivery (*p*-value < 0.001). Details of the multiple linear regression model are shown in Table 4.

**Table 4:** Details of the multiple linear regression model of the fear scores of the pregnant women

Variable	Unstandardized coefficients	SE	Standardized coefficients	*t*	*p*-value
Educational level	5.25	1.37	0.19	3.84	< 0.001
Place of residence	−3.50	1.29	−0.13	−2.72	0.007
Self-rated level of satisfaction with social life	−5.45	1.86	−0.15	−2.93	0.004
Self-rated level of satisfaction with religious commitment	8.19	2.54	0.16	3.23	0.001
Have had cesarean delivery under anesthesia	4.11	1.45	0.14	2.84	0.005
Self-declared preference for mode of delivery	−6.13	1.48	−0.20	−4.13	< 0.001

## Discussion

In this study, the fear of anesthesia for cesarean section among Palestinian pregnant women and the factors associated with fear were determined. To the best of our knowledge, this is the first study of its type in Palestine.

In this study, the majority of pregnant women expressed fear of anesthesia. In previous studies, women expressed more fear of anesthesia compared to males (Mavridou et al. 2013; Kilinc and Ozer 2017; Ruhaiyem et al. 2016). The findings of this study indicated that pregnant women who received a university education expressed a higher fear of anesthesia compared to women who received a school education only. Probably, pregnant women who received a university education could be more knowledgeable of the risks associated with anesthesia compared to less educated women (McMullan 2006).

The findings of this study showed that pregnant women who lived in rural areas expressed higher fear compared to those who lived in urban areas. In Palestine, women who live in rural areas often maintain stronger social relationships with neighbors and the surrounding society. Discussing and sharing experiences with other women who might have undergone anesthesia for cesarean delivery could have increased awareness of women about the risks associated with anesthesia. Previous studies have shown that health literacy and health information-seeking behavior affected fear of medical procedures including anesthesia (Suka et al. 2015).

In this study, self-rating satisfaction with social life was negatively associated with fear. Probably, the pregnant women who were not satisfied with their social life experienced higher levels of stress, anxiety, and fear in their lives. This could also affect their fear of medical procedures like anesthesia. Additionally, the pregnant women who have had cesarean section delivery under anesthesia expressed higher fear compared to those who did not have cesarean section delivery. Probably, this could be explained by the past/negative experiences that the women might have experienced before (Carvalho et al. 2005). Therefore, it was not surprising that women who feared anesthesia preferred normal delivery over cesarean delivery.

The findings of this study highlighted the need to address the fear of anesthesia among women scheduled for cesarean section deliveries. In this study, the majority of the women expressed fear of classic concerns reported in previous studies conducted elsewhere (Mavridou et al. 2013; Kilinc and Ozer 2017; Ruhaiyem et al. 2016; Dursun et al. 2011). It is noteworthy to mention that anesthesiologists, gynecologists and obstetricians, nurses, midwives, and other providers of antenatal and perioperative healthcare services should address the fear of anesthesia among women who might be candidates for cesarean delivery.

### Strengths and limitations

The following strengths can be considered when interpreting the findings of this study. First, this was the first study to assess the fear of anesthesia for cesarean section among Palestinian pregnant women. The findings reported in this study could be informative to providers of antenatal and perioperative healthcare services. Second, a large number of pregnant women were included in this study. Additionally, the pregnant women were diverse in terms of demographic characteristics. The large sample size and diversity of the demographic characteristics of the pregnant women should have improved the representativeness of the entire pregnant women in Palestine and the external validity of the findings. Third, the demographic variables collected in this study allowed comparing the different groups of pregnant women based on their demographic variables. Fourth, the questionnaire that was used in this study was pilot-tested for test-retest reliability and internal consistency.

On the other hand, this study had some limitations. First, the study was conducted in a cross-sectional design. Cross-sectional studies lack temporality and the ability to establish causal relationships. Although associations between the characteristics of pregnant women and fear of anesthesia were identified, no causal relationships could be established. Second, the pregnant women were recruited using a convenience sampling approach. This nonprobability sampling approach is associated with selection bias. However, the sample of pregnant women who were recruited in this study was diverse in terms of demographic characteristics like age, marital status, place of residence, educational level, and employment status. Third, the data collected in this study were self-reported by the pregnant women. Self-reported data and experiences are subject to recall and desirability bias.

## Conclusion

Fear of anesthesia was prevalent among pregnant women in Palestine. Higher fear could be predicted by education level, place of residence, satisfaction with social life, satisfaction with religious commitment, having had cesarean delivery under anesthesia, and preference for normal delivery. Anesthesiologists, gynecologists and obstetricians, nurses, midwives, and other providers of antenatal and perioperative healthcare services should consider these factors while counseling pregnant women and addressing their fear of anesthesia.

## Data Availability

All data analyzed in this study were included in the manuscript.
